# Data Empowerment of Decision-Makers in an Era of a Pandemic: Intersection of “Classic” and Artificial Intelligence in the Service of Medicine

**DOI:** 10.2196/24295

**Published:** 2021-09-10

**Authors:** Gil A Geva, Itay Ketko, Maya Nitecki, Shoham Simon, Barr Inbar, Itay Toledo, Michael Shapiro, Barak Vaturi, Yoni Votta, Daniel Filler, Roey Yosef, Sagi A Shpitzer, Nabil Hir, Michal Peri Markovich, Shachar Shapira, Noam Fink, Elon Glasberg, Ariel Furer

**Affiliations:** 1 Medical Corps Israel Defense Force Ramat Gan Israel; 2 Heller Institute of Medical Research Sheba Medical Center Tel-HaShomer, Ramat Gan Israel; 3 Department of Military Medicine The Hebrew University of Jerusalem Jerusalem Israel; 4 Planning Directorate Israel Defense Force Tel Aviv Israel; 5 Computer and IT Directorate Israel Defense Force Tel Aviv Israel; 6 Israel Veterinary Services Ministry of Agriculture and Rural Development Ramat Gan Israel; 7 Institute for Research in Military Medicine Faculty of Medicine The Hebrew University of Jerusalem Jerusalem Israel; 8 The Azrieli Faculty of Medicine Bar-Ilan University Safed Israel

**Keywords:** COVID-19, medical informatics, decision-making, pandemic, data, policy, validation, accuracy, data analysis

## Abstract

**Background:**

The COVID-19 outbreak required prompt action by health authorities around the world in response to a novel threat. With enormous amounts of information originating in sources with uncertain degree of validation and accuracy, it is essential to provide executive-level decision-makers with the most actionable, pertinent, and updated data analysis to enable them to adapt their strategy swiftly and competently.

**Objective:**

We report here the origination of a COVID-19 dedicated response in the Israel Defense Forces with the assembly of an operational Data Center for the Campaign against Coronavirus.

**Methods:**

Spearheaded by directors with clinical, operational, and data analytics orientation, a multidisciplinary team utilized existing and newly developed platforms to collect and analyze large amounts of information on an individual level in the context of SARS-CoV-2 contraction and infection.

**Results:**

Nearly 300,000 responses to daily questionnaires were recorded and were merged with other data sets to form a unified data lake. By using basic as well as advanced analytic tools ranging from simple aggregation and display of trends to data science application, we provided commanders and clinicians with access to trusted, accurate, and personalized information and tools that were designed to foster operational changes and mitigate the propagation of the pandemic. The developed tools aided in the in the identification of high-risk individuals for severe disease and resulted in a 30% decline in their attendance to their units. Moreover, the queue for laboratory examination for COVID-19 was optimized using a predictive model and resulted in a high true-positive rate of 20%, which is more than twice as high as the baseline rate (2.28%, 95% CI 1.63%-3.19%).

**Conclusions:**

In times of ambiguity and uncertainty, along with an unprecedented flux of information, health organizations may find multidisciplinary teams working to provide intelligence from diverse and rich data a key factor in providing executives relevant and actionable support for decision-making.

## Introduction

SARS-CoV-2 was first reported in Wuhan, China, by early December 2019 [1,2] and by March 11, 2020, COVID-19 was declared a pandemic by the World Health Organization [3]. To date, the global reported death toll of the disease exceeds 1.5 million individuals, while many countries continue to struggle with the pandemic and show an ascending number of cases and mortality rate [4].

Under immense uncertainty, and based on ominous models describing disease exponential expansion [5,6], Israel, like the many other countries, began preparing for the potential threat of the pandemic [7]. The Israel Defense Forces (IDF), one of the largest organizations in the country, was responsible for all health care–related aspects of its own personnel and also took action on the national front and maintained full preparedness for any of its assignments and duties—military and civilian.

One of the key understandings early during response-planning was the paramount importance of reliable data as a substrate for policy changes and decision-making [8,9]. With this mindset, the IDF surgeon general commissioned the establishment of an operational IDF Data Center for the Campaign against Coronavirus (ID3C) in March 2020. The ID3C was ordered to collect and analyze data from all venues and produce concise, data-driven, up-to-date, and relevant recommendations to guide both medical and commanding echelon policy makers.

This paper describes the assembly of the ID3C, the architecture of data utilization, and few samples of the deliverables for which the center was responsible. With much more to learn about the COVID-19 response, worldwide authorities and medical communities strive for quality data as the base for their decision- and policy-making [8-10]. We believe that sharing the lessons from our experience of creating a data center amid a raging pandemic can help other organizations to respond currently an in future to this global event and to other incidents where prompt effective action needs to be based on robust data.

## Methods

### Methods Overview

The first step of designing the ID3C was rooted in several key assumptions. We assumed that the disease will have distinct characteristics within our organization compared with the general population, mostly owing to the age of the young population and a relatively low rate of individuals with known risk factors for severe disease [11,12]. Notably, we considered this a dynamic process, as the definition of risk factors kept changing with more evidence accumulating globally at a very high pace [13]. In addition, based on reports suggesting a relatively high rate of asymptomatic individuals among young adults [14,15], we created a digital pathway to shorten the time from patient diagnosis to the completion of contact-tracing. Throughout the process, we weighted the impact of disease spread and policy modifications against the need for preparedness, and assessed the cost-effectiveness of the measures taken by using accurate data.

### Modus Operandi

**Figure 1:**
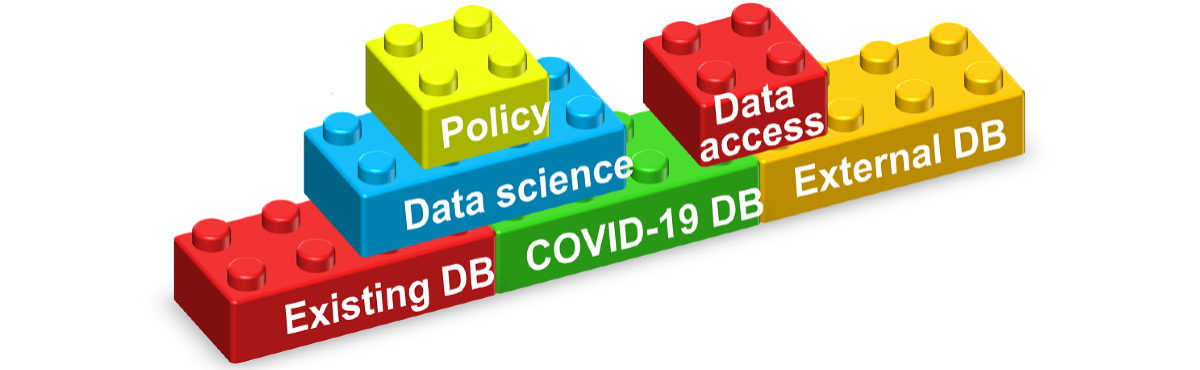
Schematic overview of the rationale for the Israel Defense Forces' Data Center for the Campaign against Coronavirus. The operation was based on the ability to bring together large databases into 1 central COVID-19 data lake. Consequently, 2 parallel efforts were conducted: the first enabled executives and commanders in the organization to gain direct access to actionable data and insights, and the second used advanced analytics to provide additional level of understanding, including using data science–based model to influence policy changes. DB: database.

First, we launched an extensive mapping of data sources and assessed the quality of data required to plan a unified data lake diverse enough to establish prediction models to act on. Next, we prioritized mirroring of trends and basic insights from aggregated data to serve as actionable information for executives. The design of a digital platform that will allow them to easily access it followed with the main vectors of action (Figure 1):

1. Harnessing existing IDF data sets: the IDF has a comprehensive digital data set of all service persons derived from decades-long data collection as part of the recruitment process of draftees, as well as electronic medical records of all military personnel. These data were merged with other “nonmedical” parameters on an individual and unit level to deepen the understanding of factors related to the risk of infection and deterioration to severe disease.
2. Construction of new COVID-19–specific information platforms and sensors.
3. Importing and merging of relevant external data provided by the Ministry of Health.
4. Unification of all data sets to 1 “data lake” and extraction of actionable knowledge followed by the use of data science tools to provide a means for pandemic management. The main attempt was to identify trends and patterns to direct the efforts to negate morbidity, especially among units critical to the competence of the organization.
5. Provision of direct data accessibility to executives: a central study objective was to improve executive-level involvement by promotion of data-driven decision-making.

### Team Assembly

The ID3C was led by 3 executives from complementary disciplines: a senior physician commanding officer, a senior intelligence officer, and a senior information technology and data officer. The ID3C comprised 5 small, specified task forces, each consisting of medical personnel, a data specialist, and a project manager. This was a first-of-its-kind military joint venture organization synergizing Medical Corps with the Computer and IT Directorate and intelligence officers. Lean task forces were responsible for gathering relevant published and collected data, providing clear insights and analyzing merged data sets, while other teams oversaw the design of “executive-friendly” dashboards.

### Data Collection

To obtain relevant insights, the IDF COVIDataLake (CDL) was created. The CDL is a fusion of existing data sets, existing COVID-19–oriented data sets, and new data sets designed by the ID3C. Existing data sets included medical records, sociodemographic data obtained from a mandatory prerecruitment survey, transportation usage, occupational characteristics, unit characteristics (ie, unit under quarantine conditions and age distribution), service characteristics (ie, daily attendance, close encounter with civilians, etc). These data helped estimate interactions among particular individuals once a suspicion of infection arose. Newly formed COVID-19–oriented data sets included the following: laboratory-based COVID-19 reverse transcription polymerase chain reaction (RT–PCR) test results of the IDF; central command and control platform, which documented all suspected, quarantined, and confirmed cases, as well as all registration of all calls to the call center with structured fields to report on symptoms and exposure to confirmed patients with COVID-19; and special data annotations to highlight quarantined and hospitalized personnel.

Based on the concept of the triple T (“trace, test, treat”) [16,17], throughout the ID3C workflow, a system of surveys was developed to collect data deemed essential for early identification of suspected individuals; these included a habits and baseline risk factors survey and a daily survey with a single “yes/no” question regarding exposure and symptoms. For those who had an indication of exposure or symptoms, a more detailed survey was prompted automatically.

To improve the accuracy and efficiency of epidemiological investigations, a designated web-based platform designed by public health officers was introduced where all data from conducted epidemiological investigations were maintained in a structured database.

Lastly, as national-level data collection improved drastically, several sources of data were found to be relevant for the IDF, including daily reports of “hot zones”; a national patients registry elaborating on symptom distribution and disease severity by age; public symptoms surveys conducted through open-source apps, health maintenance organizations, and the Ministry of Health [18]; and risk models for progression to severe disease developed by the “Clalit health services” [19].

### Data Security and Ethics

The CDL was treated as a medical database in accordance with national regulations. Data collection and analysis were approved by institutional review board of the IDF Medical Corps (submission 2082-2020).

### Data Analysis and Predictive Data Science

The proportions of soldiers treated with respiratory symptoms but without a COVID-19 diagnosis were compared between units. The annotation of theses visits was available using natural language processing techniques to search through medical records and seek signs and symptoms related to COVID-19 (eg, shortness of breath, fever, cough, anosmia, and ageusia). Units were updated once an alarming increase in such visits was evident as an early sign for a possible concealed cluster of COVID-19 cases. Additionally, rates of individuals at risk for severe disease were calculated on the basis of computerized exploration of the medical records for a list for pre-existing medical conditions and treatments, in accordance with updated literature concerning risk factors for severe COVID-19. Shortlists of all at-risk individuals at the unit level were provided to the commanding level daily to reconsider the importance of the individual in terms of the unit’s preparedness and functionality.

Machine learning models were used to optimize the IDF’s queue for RT–PCR examination for COVID-19. The data points were split into three sets: training, testing, and validation. The validation set comprised 15% of the most recent data points. This set was used to estimate the generalization error of the model. A chronological partition was chosen so as not to underestimate the generalization error: the data were observably chronologically irregular, stemming from the rapid change in the policies, guidelines, and spread of the disease. To deal with these trends, 2 additional sources were incorporated into the data set. First, cities and towns with high contagion counts were recorded daily from public data provided by the Ministry of Health. This allowed us to approximate the suspects’ risk of exposure stemming from their city of residence. Second, the number of quarantined personnel in the individual’s unit was calculated from the IDF’s daily attendance record. This allowed us to account for the current extent of quarantine and policies mandated in the individual’s close work environment. Including these 2 variables in the data set led to an observable improvement in the models’ performance. The testing set was used by the models’ internal optimization procedure to evaluate intermediate trained models and to tune the models’ parameters. The training set was used to train the intermediate models. Models were evaluated in accordance with the intended use case: prioritizing the RT–PCR testing queue, with the goal of increasing the proportion of positive cases among those tested. Given the typical daily laboratory capacity and queue length, the score used to evaluate the models was the proportion of true-positive cases among 10% of cases with the highest predicted risk score. This is essentially the pretest probability of the model, with a dynamic threshold (ie, the first predicted high-risk decile of the test queue, in contrast with a constant risk score threshold). Data handling and utilization were conducted using database SQL (Toad for Oracle, version 18c; Quest Software Inc) and SAS (version 9.4, the SAS Institute). Python (version 3.6, Python Software Foundation) was used throughout the study. Preprocessing and exploratory data analysis were conducted with Pandas and Seaborn Python packages. The Scikit-learn Python package was used for training and evaluation of models.

## Results

Nearly 300,000 responses for daily questionnaires were recorded by the end of July 2020 with compliance variance from 50% of all personnel in some periods to less than 10% in most units routinely.

### Data Sharing Platforms for Executives

**Figure 2:**
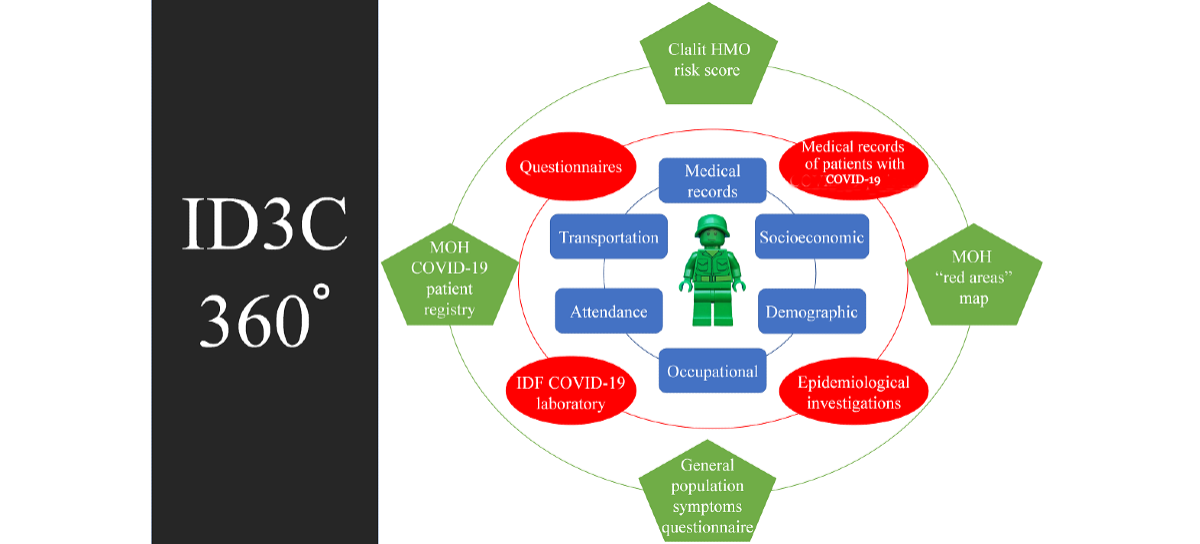
ID3C 360° individual data components. The unique datasets collected during the operation of the ID3C included three layers of data in particular: data set available routinely in the IDF, including medical records, a human-resources data set with demographic and occupational characteristics, and daily attendance and logistic data including transportation usage; a new data set designated to collect COVID-19–related data such as laboratory findings, epidemiological investigations' outputs, designated surveys, and confirmed cases' medical records; and external data sets from government agencies, other health organizations, and open-source publications on broad population surveys. ID3C: Israel Defense Forces’ Data Center for the Campaign against Coronavirus; IDF: Israel Defense Forces; HMO: health maintenance organization; MOH: Ministry of Health.

**Figure 3:**
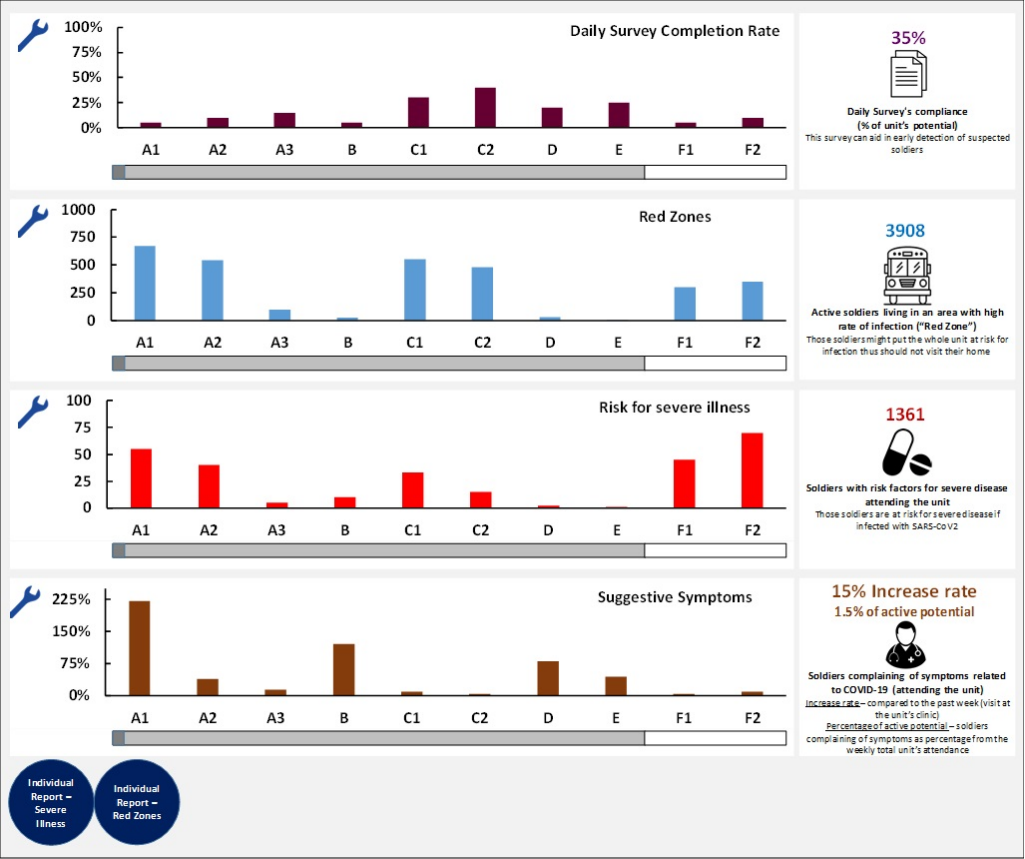
Daily updated main display of the Israel Defense Forces’ Data Center for the Campaign against Coronavirus digital platform designed for commanders' use. This includes the daily survey completion rate, which is the proportion of the unit’s potential (purple, top panel); red zones, which refers to active soldiers living in an area with a high rate of infection (blue, second panel from the top); risk for severe illness, which refers to soldiers with risk factors for severe disease if infected with SARS-CoV-2, who attend the unit (red, third panel from the top); and suggestive symptoms, which is the proportion of on-duty soldiers presenting to unit's clinic for symptoms related to COVID-19 (increase rate refers to the increase in visits compared to the past week; percentage of active potential refers to soldiers complaining of symptoms as a percentage from the weekly total unit’s attendance; brown, bottom panel).

One centralized digital platform was designed to provide every unit and senior commanders the following daily updated information about their unit (Figure 2):

1. Identifying individuals living in “hot zones”: indeed, analysis conducted using military epidemiological data revealed that 56% of disease contraction was outside of the military base or activity.
2. Identifying individuals attending the unit, who are at risk for severe disease: a decline of ~30% was observed in the attendance of such individuals after the implementation of this tool (Figure 3).
3. Notification on units with alarming clusters of respiratory complaints.

### Data Sharing Platforms for Medical Personnel

Unit medical staff had access to a daily updated dashboard, which contained the following information:

1. Notification of individuals at high risk for severe disease: direct communication between the ID3C and units’ primary physicians was facilitated with respect to extremely-high-risk individuals by using the Clalit algorithm.
2. Alert on individual cases that form a part of clusters presenting with respiratory symptoms, allowing physicians to revisit cases if an alarming number of patients with suspicious symptoms presented at a specific clinic.
3. Medical indicators from surveys promoted proactive summoning of individuals reporting suspicious symptoms and exposures before they approached the clinic, and those found to be at substantial risk were directed to undergo a nasopharyngeal swab test.

### Optimization of the Queue for Laboratory-Based COVID-19 Testing

For each individual in the examination queue, which included thousands of individuals at peak periods, the model produced a risk score, which was used to prioritize the predicted high-risk suspect. Based on CDL, a prediction model was trained and tested on 10,428 examinations and validated on additional 1698 examinations. The model indicated daily the potential top 10% high-risk suspects with a very high pretest probability and prioritized the processing of their nasopharyngeal swabs. This optimization process resulted in a high true-positive rate of 20% from among the total examination results processed, which was more than twice as high (2.28%, 95% CI 1.63%-3.19%) as the baseline true-positive rate (8.8%) without using the model recommendation for the same period.

## Discussion

### Principal Findings

**Figure 4:**
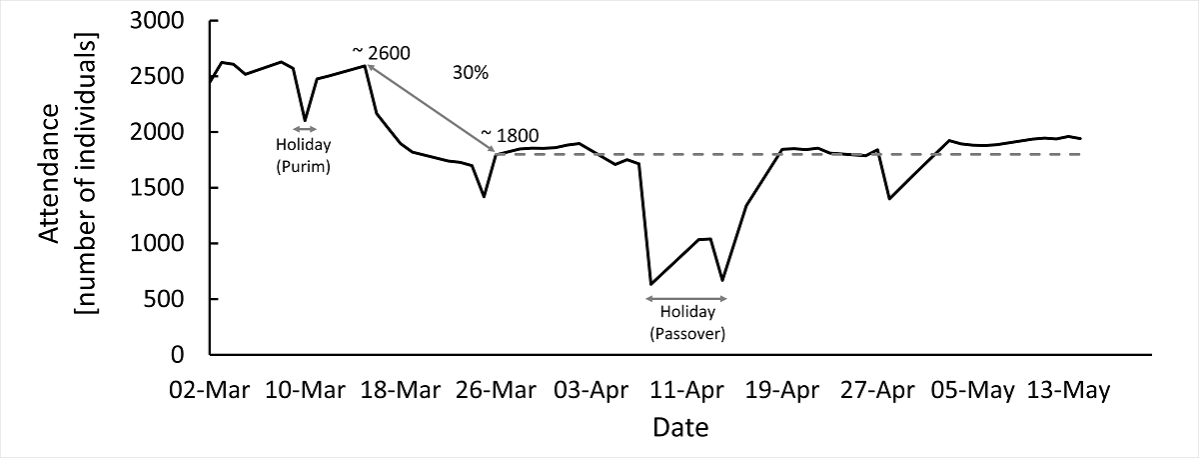
The attendance of individuals deemed at high risk for severe disease in the Israel Defense Forces' units, between March 2 and May 13, 2020. The graph shows a sharp decline in attendance with the implementation of the designated unit-specific tool, allowing to monitor the individuals at risk for severe disease.

We report here the origination of a COVID-19 dedicated response by the IDF with the assembly of the ID3C. The multidisciplinary team utilized existing and newly developed platforms to collect and analyze large amounts of information for each individual in the context of SARS-CoV-2 contraction and infection. With nearly 300,000 responses for daily questionnaires merged into 1 data lake, we could identify high-risk individuals, and we observed a 30% decline in their attendance to their units (Figure 4). Moreover, we introduced an optimization model for the queue for laboratory-based COVID-19 testing and obtained a high true-positive rate, which was twice as high as the baseline rate (20% vs 8.8%, respectively).

Both health care providers and leaders are in the midst of a global crisis, which is characterized by extreme uncertainty and unprecedented flux of information. In this “infodemic” and pandemic, policy makers pursue trustworthy measures to help them make strategic decisions in response to the threat [20,21].

A call for innovative, modern methods of rapidly collecting and processing data has been made by countries and organizations around the world from the beginning of the pandemic. Taiwan—a neighboring country to China, which was one of the first countries to be affected by the pandemic—took rapid action by merging the borders and customs databases with their national medical database to link clinical symptoms with flight and travel history.

As early as January 2020, Sun et al [22] and Qin et al [23] used social media searches to track the trends and spread of COVID-19 through China. Indeed, web-based discussions on social media platforms prompted an international group of rheumatologists to create the COVID-19 Global Rheumatology Alliance [24]. This alliance created a web-based registry in which clinicians and researchers around the world could share their knowledge and findings. The American Academy of Dermatology, American College of Surgeons, American Academy of Orthopedic Surgeons, Spanish Neurological Society, and others have also created specialized registries to track and analyze relevant data in their fields [25,26].

Many other methods have been implemented, such as web-based surveys to detect the spread of the disease in the population [27,28], and the use of machine learning techniques to quickly and reliably test for COVID-19 by private firms such as *Infervision*, *Diagnostic Robotics*, and others [29].

This atmosphere of uncertainty has many similarities to other emergency situations, especially those concerning security, where leaders depend on intelligence officers to provide them with data and estimations. We describe here the prompt establishment of a unique organizational structure set to provide live intelligence, using a synergism of “conventional” military intelligence methods, medical knowledge, and research, as well as data-driven artificial intelligence platforms. The center was based on multidisciplinary teaming, utilization of data, and leaning on digital platforms for providing accurate, relevant, and comprehensive information to executives. The novelty in this process covers multiple aspects and was catalyzed by the intensity of the challenge, and in fact fulfilled many of the perspectives that health care professionals had envisioned in the last decade with respect to the era of information, personalized medicine, and digital health [30-32].

Identifying the engagement of medium-tier commanders as the key to maximize the chance to make an impact dictated our pivotal effort to design tools that would not merely end up with data analysis but would be delivered almost immediately as actionable data, to affect their daily routine. Similar to the approach adopted by others, we found the delivery of accurate and timely knowledge translation an inevitable effort in light of the surging pandemic [33].

The tools used in the ID3C were designed to promote the prevention of contagion, earlier detection of disease clusters, and better control of at-risk individuals. Other data-driven tools were actually developed in response to an emerging challenge with an extreme shortage of RT–PCR testing and provided optimization to make better use of existing testing methods [34], using computer-aided prioritization of examinations, thus increasing the proportion of positive test results and decreasing the mean diagnosis time of positive cases.

Notwithstanding, the experience of collecting large amounts of data provided valuable insights into the importance of careful planning of the delicate architecture and the quality assurance measures that are a “must-have” in this type of endeavor. Data science and machine learning are very common “buzzwords” in the discussion about the health care revolution [35-37], but one should be very cautious to not overload expectations from these set of tools, especially when the data are gathered at different paces and through different channels. Moreover, this emphasizes the need for local and global medical data sharing among organizations and countries, which would be key for enabling the utilization of emerging computational proficiencies and potentially shed light on clinical interpretation of events, thereby accelerating the discovery and validation of promising interventions [8,38-40].

A centralized medical intelligence arm, where public health officers, physicians, and data experts work together to construct the process of data collection, analysis, and reflection, was found to be very efficient and had a true synergistic value in our experience. The ability to combine the technological arm that designs and broadcasts the different applications for data collection had an additional value and enabled swift changes guided by real-time field feedback. This is further outlined when compared with conventional public health methods primarily based on manually collected data and the tendency to work in isolated professional silos, limiting prompt data analysis and action required to effectively respond to the current COVID-19–imposed challenges [41]. The agility in producing the right set of tools for constantly changing circumstances is crucial for successful efforts in the long term, as the pandemic changes and evolves.

A vital consideration we felt obligated to maintain was the high standard of medical confidentiality, highly ethical standards of data science research, and privacy of data. Despite the urgency and the need to deliver data, we insisted on and maintained 2 separate routes of data flow: one for medical personnel and the other for nonmedical executives. This allowed us to provide meaningful alerts and information at the executive level while keeping the medical staff at the center of the decision-making process, being able to consult judiciously with a very broad clinical overview, and simultaneously not breaching any medical privacy standard.

### Limitations

We experienced several difficulties that are worth mentioning in this context. First, we saw very modest compliance rates to the extensive survey effort that we initiated, especially as the timing of broadcasting of the full-scale survey coincided with a sharp decline in cases and an increase in survey compliance with the substantial surge in cases. As with other noncompliance issues in medicine, it is our belief that for the time being, a cultural change is the key to deliver a discernible change. Given its potential effectiveness in the early identification of suspicious domains, a survey should be considered a routine procedure throughout the pandemic. Second, the current pandemic is very dynamic, and the nature of disease spread was different between the 2 main pandemic periods we observed. The knowledge base to understand this specific new infection is lacking and evolves constantly, and this might have a substantial effect on any mathematical model, thus necessitating cautious evaluation of models based on concurrent data. To ensure our tools are valid, we continuously evaluated the yield and accuracy of our models and modified the included parameters to adapt to the changing circumstances. Lastly, despite our extensive effort in building data-driven tools, it is of paramount importance to recall their role as advisory tools and highlight the central role of human judgment in integrating professional, medical, and organizational considerations at all times.

### Conclusions

In conclusion, we described the assembly of a specialized multidisciplinary team weighing on diverse, rich data and advanced analytics to assist decision-makers with formulating policies in the context of the unexpected and unfamiliar chain of events throughout the COVID-19 pandemic. With anticipated disease spread, we believe that additional collaborative initiatives are in place to largely utilize accumulating local and global data in an attempt to develop impactful interventions.

## Abbreviations

CDL: IDF COVIDataLake
ID3C: IDF Data Center for the Campaign against Coronavirus
IDF: Israel Defense Forces
RT–PCR: reverse transcription polymerase chain reaction
